# Dietary Cholesterol Requirements of Large Red Swamp Crayfish (*Procambarus clarkii*)

**DOI:** 10.1155/2023/6697222

**Published:** 2023-12-05

**Authors:** Juan Tian, Wenfu Xiao, Weihua Gao, Jianmin Zhang, Liangzi Xu, Mingzhu Li, Hongwei Liang, Ningning Xie, Lixue Dong, Jie Li

**Affiliations:** ^1^Yangtze River Fisheries Research Institute, Chinese Academy of Fishery Sciences, Wuhan 430223, China; ^2^College of Animal Science, Yangtze University, Jingzhou 434024, China; ^3^College of Agriculture, Ludong University, Yantai, China; ^4^Hubei Fisheries Science Research Institute, Wuhan, Hubei 430071, China

## Abstract

To investigate the dietary cholesterol requirements of large red swamp crayfish (*Procambarus clarkii*), crayfish (initial body weight: 13.49 ± 0.22 g) were hand-fed six diets containing 2.47 (C0), 4.27 (C1), 6.80 (C2), 8.77 (C3), 11.74 (C4), and 14.24 (C5) g/kg cholesterol. After 8 weeks of feeding, the maximum weight gain rate and specific growth rate occurred in group C4. The lowest feed conversion ratio was observed in group C3. Total flesh percentage increased significantly by 15.33% in group C2 compared to group C0. The increase in dietary cholesterol resulted in significant quadratic trends in concentrations of crude protein and lipid in muscle and whole body; cholesterol and free fatty acid in hemolymph, hepatopancreas, and muscle; activities of lipase and amylase in hepatopancreas and intestine; and total antioxidant capacity and catalase activity in hepatopancreas. Group C3 experienced a noteworthy increase in hemolymph glucose and total protein content compared to group C0. Additionally, malondialdehyde content and superoxide dismutase activity in hepatopancreas displayed significant linear and quadratic trends. The optimal dietary cholesterol level for large *P. clarkii* is between 7.42 and 10.93 g/kg, as revealed by the quadratic regression analysis.

## 1. Introduction

Cholesterol (C_27_H_46_O, CHO), is a cyclopentane polyhydrophenanthrene derivative that is commonly present in free form or as cholesteryl esters in a variety of animals, but is virtually absent in plants. Cholesterol plays an essential role in maintaining the fluidity of cell membranes [1], and is a precursor for the synthesis of some steroid hormones, including sex, decidual, and adrenocortical hormones [2, 3]. In addition to its other functions, cholesterol is involved in the synthesis of bile acids and vitamin D_3_, coordinates nutrient metabolism, and indirectly contributes to the normal functioning of the nervous system [4]. Although fish can synthesize cholesterol from acetic acid [2], crustaceans cannot due to the intermediate reaction from farnesyl pyrophosphate to squalene is blocked [2, 5]. Crustaceans lack a gallbladder which hinders their ability to convert excess cholesterol to bile acids by secreting bile. In crustaceans, previous studies have shown that, cholesterol plays a crucial role as an essential nutrient associated with shell molting, reproduction, and growth [2]. Intestinal cells absorb exogenous cholesterol which induces the formation of cholesteryl esters at the endoplasmic reticulum. These esters, along with triglycerides, phospholipids, and a small amount of free cholesterol, are then assembled into chylomicrons [2]. After absorption, cholesterol is mainly transported to the subcutaneous tissues, gonads and hepatopancreas. It is secreted into the lymphatic circulation through the basement membrane, where it participates in the regulation of various life activities. Excess cholesterol is mainly stored in hepatopancreas as cholesteryl esters [2, 6].

The cholesterol content of fish oil and fish meal is typically in the range of 0.1%–0.7% [7]. The increasing cost of animal protein sources has led to the increased utilization of plant protein sources in the crustacean diets. This indicates that the amount of dietary cholesterol in crustaceans is relatively low. Consequently, cholesterol supplementation is necessary to fulfill the demands of regular physiological metabolism. Crustaceans may experience slow growth and increased mortality due to the cholesterol deficiency. For example, dietary cholesterol deficiency diminished the growth performance of *Cherax quadricarinatus* [8], caused *Scylla serrat* to take longer to molt [9], and reduced survival [10]. However, dietary supplementation with excess cholesterol increased the number of molts in *Penaeus japonicus* [11] and inhibited the growth of *Macrobrachium rosenbergii* [12] and *Penaeus monodon* [13]. As such, it is essential to maintain optimal levels of dietary cholesterol to enhance the growth performance and feed utilization of crustaceans. The feed efficiency of *Litopenaeus vannamei* was improved by supplementing the diet with 1.90 g/kg cholesterol; and using phytosterol as a partial replacement for cholesterol [14]. The optimal dietary cholesterol requirement for *Penaeus japonica* was found to be 5.00 g/kg. Furthermore, the dietary cholesterol requirements showed a decreasing trend with an increase in feeding rate [12]. The dietary cholesterol content of 11.2 g/kg resulted in the highest weight gain rate (WGR) for *Macrobrachium nipponense* [15]. *Scylla paramamosain* obtained the maximum WGR when the dietary cholesterol content was 11.1 g/kg, and the total body cholesterol content increased as the dietary cholesterol content increased [10]. The cholesterol requirements of crustaceans vary based on the factors such as species, growth stage, evaluation index, and the source, content, and utilization of cholesterol and phospholipids present in the experimental diets. Most crustaceans have dietary cholesterol requirements in the range of 2.0–10.0 g/kg.

Red swamp crayfish (*Procambarus clarkii*), commonly known as crayfish, belongs to the Phylum Arthropoda, Crustacea, Decapoda, and *Procambaridae*, and is native to North America [16]. *P. clarkii* was introduced into China in the 1930s; and has been widely consumed in China in the recent years [17]. In 2022, the annual production of crayfish was 2.89-million tons, ranking the fourth in freshwater aquaculture and being the first of freshwater shrimp in China [18]. Therefore, the increasing demand of crayfish has given great impetus to the development of its feed industry. Our previous study has reported the requirements of dietary fat [19], protein [20], calcium [21], and phosphorus [16], as well as the optimal lipid sources for crayfish [22]. The dietary cholesterol requirements of juvenile *P. clarkii* (initial body mass: 4.4 ± 0.1 g) was estimated to be 5.4 g/kg [23]. It is noteworthy that the diets of large *P. clarkii* reared in captivity are largely devoid of animal ingredients, which limits their intake of cholesterol and affects their growth and reproductive capacity. In view of the relatively high cost of commercial high-purity cholesterol, it is imperative to more accurately define the cholesterol requirements of *P. clarkii* at different growth stages to regulate feeding costs. The present study aimed to investigate the dietary cholesterol requirements of large *P. clarkii* (initial body weight: 13.49 ± 0.22 g) in light of the previous studies, with particular emphasis on the accumulation of cholesterol in tissues and its impact on muscle growth and the health of the hepatopancreas. The results of this study could serve as a scientific basis for the future development of the *P. clarkii* feed industry.

## 2. Materials and Methods

### 2.1. Ethics Statement

Red swamp crayfish are widely cultivated in China and are not listed as an endangered or protected species. All animal care and use procedures were approved by the Institutional Animal Care and Use Committee of Yangtze River Fisheries Research Institute (according to YFI 2018–40 dated July 20, 2018). Prior to allocating to the tanks and blood sampling, crayfish were anesthetized with 30 mg eugenol/L water to minimize suffering. In this experiment, they were anesthetized to death with 60 mg eugenol/L water before body and tissue sampling.

### 2.2. Experimental Diets

Proteins were sourced from fish meal, soybean meal, gluten meal, and peanut meal, while carbohydrate were sourced from flour and corn starch, and lipids were sourced from fish oil and corn oil. Cholesterol was acquired from Shanghai Yuanye Biotechnology Co., Ltd. (Shanghai, China). The purity of the acquired cholesterol was 95%. The cholesterol-supplemented groups added either 3, 6, 9, 12, or 15 g/kg supplemented with microcrystalline cellulose to balance the doses, while the control group added 0 g/kg. The cholesterol content of the six diets was measured and recorded as 2.47 (control group), 4.27, 6.80, 8.77, 11.74, and 14.24 g/kg, respectively, and denoted as C0, C1, C2, C3, C4, and C5.  Table 1 shows the dietary formulations. All the raw materials were weighed accurately, milled through a 0.25-mm sieve, and mixed using the step expansion method. The pellet feed with a diameter of 2 mm was manufactured utilizing an F-26 twin-screw extruder (Huagong Optical Mechanical and Electrical Technology Co., Ltd., Guangzhou, China). After the curing process for 20 min at 90°C, the feed underwent a drying process for 2 hr at 60°C with a crawler feed dryer (DW Series Mesh-belt Dryer, Suzheng Drying Equipment Co., Ltd., Changzhou, China). The dried feed was then crushed into cylindrical pellets with diameters ranging from 3 to 4 mm, stored in a refrigerator at −20°C until use.

### 2.3. Experimental Shrimp and Feeding Management

The culture experiment was conducted in an indoor micro-water culture system at Yangtze River Fisheries Research Institute, Chinese Academy of Fishery Sciences. Each culture tank (110 cm × 80 cm × 30 cm) was equipped with protection tubes to prevent cannibalism. Crayfish were purchased from the seedling breeding farm (Qianjiang, China) and fed the control diet for 2 weeks to adapt to the experimental environment and diet. Following a 24-hr period of fasting, crayfish with uniform size (initial body weight: 13.49 ± 0.22 g), complete chelate feet and good vitality were selected and randomly divided into 18 tanks; each tank contained 18 crayfish and was used to rear them in the micro-water culture system. At the same time, 20 shrimp were randomly selected and stored at a temperature of −40°C in a refrigerator to determine their initial proximate composition. The feeding rate was adjusted as required based on the feeding conditions, water temperature, and other environmental factors. Bait residues were collected 2 hr after feeding and stored at a temperature of −20°C in a refrigerator. At the end of the feeding trial, feed residues were dried, weighed, and used to calculate the total feed weight. Every day, each tank was gradually refilled with 300 L of fresh water at a microflow rate of 30 L/hr. Daily records were kept of water temperature, feed consumption, and the number of deceased crayfish. Feces were aspirated every morning. Throughout the experiment, the water temperature was 23–28°C, ammonia nitrogen was ≤0.05 mg/L, dissolved oxygen was ≥5 mg/L, and pH was 8.3 ± 0.1. The feeding trials were conducted for a period of 8 weeks.

### 2.4. Sample Collection

At the end of the feeding period, crayfish were counted and weighed individually after a 24-hr fast to calculate their growth performance. Two crayfish per tank were randomly selected and freeze-dried for whole body nutrient composition analysis. In addition, nine crayfish were selected from each tank and hemolymph was collected from the pericardial cavity using a 1-mL syringe. The hemolymph was placed in a 1.5-mL centrifuge tube at 4°C for 4 hr, then centrifuged (4°C, 14,400×*g*, 20 min) to collect the supernatant. The supernatant was stored in a refrigerator at −80°C to detect serum biochemical indices. The crayfish was then dissected, and the hepatopancreas, abdominal muscles, and muscles of the first chelopod were removed and weighed to calculate the hepatosomatic index (HSI) and total flesh percentage (FP). The weighed muscles, hepatopancreas and intestines were placed in zip-lock bags and frozen at −40°C to determine other indices. The hepatopancreas (0.5 cm × 0.5 cm × 0.5 cm) of two crayfish per tank was cut and placed in 4% paraformaldehyde for 24 hr, then stored in 70% ethanol until hematoxylin–eosin (H&E) staining.

### 2.5. Indices Measurement

#### 2.5.1. Determination of Proximate Composition

The moisture content of the diets was determined by drying at 105°C for 24 hr. The freeze-drying method was used to determine the moisture content of whole shrimp and muscle (CHRIST freeze-drying machine, Germany). Crude protein content was determined by the Kjeldahl nitrogen method. Crude fat content was determined by the Soxhlet extraction method. Ash content was determined by the muffle furnace combustion method at 550°C. Energy level was determined by the direct combustion in an adiabatic bomb calorimeter (SDC311, Hunan Sundy Science and Technology Development Co., Ltd., Changsha, Hunan Province, China).

#### 2.5.2. Determination of Serum Biochemical Indexes

The contents of glucose (GLU), total protein (TP), and albumin (ALB) were determined by the hexokinase method, polyurea method, and BCG method, respectively. Aspartate aminotransferase (AST) and alanine aminotransferase (ALT) activities were determined by the LDH-UV and MDH-UV methods, respectively. An automated biochemical analyzer (BX-3010, Sysmex Corporation, Tokyo, Japan) was used to determine the above serum biochemical indices. Reagents used were purchased from the Sysmex Corporation.

#### 2.5.3. Determination of Digestive Enzyme Activities

The hepatopancreas and intestine samples were accurately weighed and nine times the volume of normal saline (0.9%) was added to each sample. The homogenate was homogenized on an ice-water bath for 30 s to obtain 10% homogenate. After centrifugation (3,000×*g*, 10 min, 4°C), the supernatant was collected for the assay. Casein was used as a substrate to determine protease activity in hepatopancreas and intestine by the Folin reagent method [21]. Lipase activity was determined using the methyl halogen substrate method assay (Cat. No.A0-2-1). Amylase activity was determined by the starch iodine chromogenic assay (Cat. No. C016-1-1). The protein content in the supernatant was determined by the Coomassie bright blue assay (Cat. No. A045-3). The definition of digestive enzyme activity was based on our previous study [21]. All kits were purchased from the Nanjing Jiancheng Bioengineering Institute (Nanjing, China).

#### 2.5.4. Determination of Lipids Content in Serum, Hepatopancreas, and Muscle

Hepatopancreas, muscle, and diet (0.5 g) were accurately weighed. Nine times the volume of precooled anhydrous ethanol at 4°C was added to diet and hepatopancreas, and nine times the volume of precooled phosphate buffer (0.1 M, pH 7.4) at 4°C was added to muscle. Mechanical homogenization was performed in ice water for 1 min. During the homogenization process, the rotation speed of the homogenizer was ensured to be consistent with the homogenization time of each sample. After centrifugation (3,000×*g*, 10 minus, 4°C), the supernatant was collected for subsequent determination. The levels of total cholesterol (T-CHO), triglycerides (TG), high-density lipoprotein cholesterol (HDL-C), low-density lipoprotein cholesterol (LDL-C), and free fatty acid (NEFA) were determined by the CHOD-PAP method (Cat. No. A111-2-1), GK-GPO-POD method (Cat. No. A110-2-1), two-reagent direct method (Cat. No. A112-1-1), two-reagent direct method (Cat. No. A113-1-1), and ACS-PAP method (Cat. No. A042-2-1), respectively, using the reagent purchased from the Nanjing Jiancheng Bioengineering Institute.

#### 2.5.5. Determination of Antioxidant Indexes of Hepatopancreas

The pretreatment of the samples was the same as the pretreatment of the samples in which the digestive enzyme activity was measured. Total antioxidant capacity (T-AOC) was determined by the colorimetric method (Cat. No. A015-1) and malondialdehyde (MDA) content by the thiobarbituric acid (TBA) method (Cat. No. A003-1). Catalase (CAT) and superoxide dismutase (SOD) activities were determined by the ammonium molybdate method (Cat. No. A007-1-1) and water-soluble tetrazole salt 1 (WST-1) method (Cat. No. 301), respectively. All kits were purchased from the Nanjing Jiancheng Bioengineering Institute.

#### 2.5.6. Hepatopancreas Section

Histological features were observed and photographed under a light microscope (OLYMPUS DP73, Japan) following gradient dehydration with ethanol, paraffin embedding, sectioning (LongerPump Yzⅱ25, 5 *μ*m, UK), H&E staining, and neutral gum sealing. According to Smith et al. [24], crustacean hepatopancreatic cells are characterized into four types: embryonic cells (E cells), which maintain their differentiation potential; secretory cells (B cells), which secrete digestive enzymes; absorptive cells (F cells), which are responsible for absorbing nutrients and synthesizing proteins; and storage cells (R cells), which store energy substances. The hepatopancreas, stained with H&E, exhibits giant E cells with nuclei that are distinguishable from the other three cells: F cells, similar to E cells, lack nuclei; B cells contain vacuoles; and R cells possess nuclei that are dark red in color.

### 2.6. Data Analysis

Statistical analysis was performed using SPSS 26.0 (IBM Corp. Released in 2019. IBM SPSS Statistics for Windows, version 26.0. Armonk, NY, USA: IBM Corp.). Prior to one-way analysis of variance (ANOVA), the collected data were checked for Levene's test to ensure normality of distribution and homogeneity of variance. In addition, all data were further analyzed for the linear and quadratic effects of increasing dietary cholesterol levels by orthogonal polynomial contrasts. Mean differences between treatments were compared using Tukey's range test at a significance level of *P* < 0.05. Figures were obtained from Origin2019 (OriginLab Company, Northampton, MA, USA).

## 3. Results

### 3.1. Effects of Dietary Cholesterol Level on Growth Performance of *P. clarkii*

**Figure 1:**
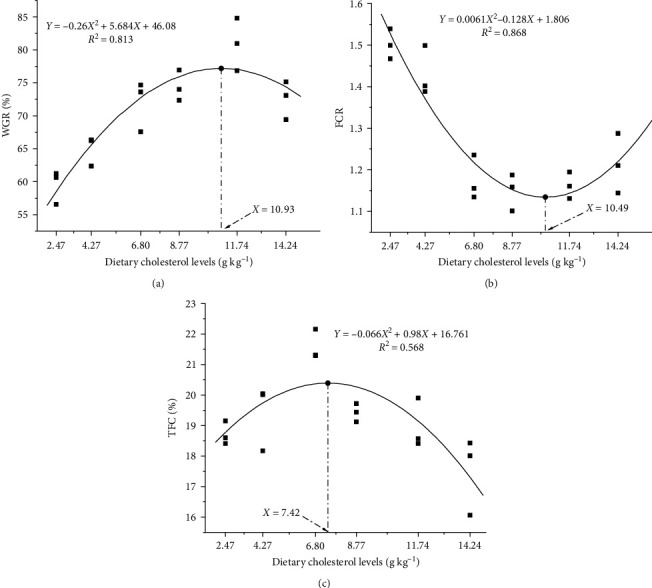
Quadratic curve analysis of the relationship between dietary cholesterol level and weight gain rate (a), feed conversion ratio (b), and total flesh percentage (c) of *P. clarkii*.

As the dietary cholesterol level increased, the crayfish exhibited a significant quadratic trend in WGR, specific growth rate (SGR), feed conversion ratio (FCR), abdomen flesh percentage (AFC), clawfoot flesh percentage (CFC), total flesh percentage (TFC), protein efficiency ratio (PER), and protein deposition rate (PDR) (*P* < 0.05). Compared to the group C0, WGR and SGR in group C4 were significantly increased by 35.98% and 27.71%, respectively (*P* < 0.05). The FCR of groups C2–C5 was significantly lower than that of the C0 group (*P* < 0.05). Group C2 demonstrated significantly higher AFC, CFC, and TFC values compared to group C0 (*P* < 0.05). In fact, TFC in group C2 was 15.33% higher than that of group C0 (*P* < 0.05). The group C3 exhibited the highest values for both PER and PDR, significantly higher than those of the C0 group (*P* < 0.05). The groups showed no significant effect on SR and HSI due to dietary cholesterol level (*P* > 0.05) (Table 2). The study conducted a regression analysis with WGR, FCR, and TFC of *P. clarkii* as dependent variables (*Y*) and dietary cholesterol level as the independent variable (*X*). The analysis revealed the optimal dietary cholesterol level to be 10.93, 10.49, and 7.42 g/kg, respectively (Figure 1).

### 3.2. Effects of Dietary Cholesterol Level on the Proximate Composition of *P. clarkii*

As the dietary cholesterol level increased, there was a significant quadratic trend in the crude protein and lipid content of the whole body and muscle (*P* < 0.05). In comparison to group C0, group C2 demonstrated significantly higher crude protein levels in the whole body, while group C5 showed significantly higher crude protein levels in the muscle (*P* < 0.05). In the cholesterol-added groups, both the whole body and muscle showed a significant decrease in crude lipid content compared to the C0 group (*P* < 0.05). There were no significant differences in moisture and ash content of the whole body and muscle, or in the moisture content of hepatopancreas among the groups (*P* > 0.05). Crude lipid content in the hepatopancreas showed a significant linear trend (*P* < 0.05), with an increase of 13.80% and 18.79% in groups C4 and C5, respectively, as compared to the group C0 (*P* < 0.05) (Table 3).

### 3.3. Effects of Dietary Cholesterol Level on Lipids Contents in Serum, Hepatopancreas, and Muscle of *P. clarkii*

With an increase in dietary cholesterol level, significant quadratic trends were observed in the concentrations of T-CHUO, HDL-C, LDL-C, and NEFA in serum, hepatopancreas, and muscle (*P* < 0.05). Additionally, TG contents in hepatopancreas and muscle showed significant linear and quadratic trends (*P* < 0.05).

In serum, HDL-C content showed a significant increase in the groups with added cholesterol compared to group C0 (*P* < 0.05). The maximum values of LDL-C and NEFA contents were observed in group C4 and group C3, respectively. Significant increases in T-CHO content were observed in the groups C4 and C5 as compared to group C0 (*P* < 0.05). Except for group C2, TG content was significantly increased in added cholesterol groups as compared to the group C0 (*P* < 0.05).

In hepatopancreas, HDL-C and LDL-C contents showed the minimum levels in group C3 and group C4, respectively (*P* < 0.05). The concentration of NEFA was significantly higher in group C4 than in group C0 (*P* < 0.05). Groups C4 and C5 showed a significant increase in T-CHO content, while groups C3–C5 showed a significant increase in TG content compared to the group C0 (*P* < 0.05).

In the muscle, there was a significant increase of 29.57%, 31.48%, and 29.50% in T-CHO, TG, and HDL-C content, respectively, in group C5 compared to group C0 (*P* < 0.05). No significant difference was found in LDL-C content (*P* > 0.05). Group C2 showed the highest concentration of NEFA (*P* < 0.05) (Table 4).

### 3.4. Effects of Dietary Cholesterol Level on Serum Biochemical Indices of *P. clarkii*

With the increased dietary cholesterol level, no significantly linear or quadratic trend of variation was observed in the contents of TP and ALB, or AST activity, while GLU content and ALT acidity showed an significant linear and quadratic trends in serum (*P* < 0.05).

Group C3 had a significant increase in GLU and TP content compared to group C0 (*P* < 0.05). The activities of AST and ALT were significantly higher in groups C2 and C4, respectively, compared to the group C0 (*P* < 0.05). There was no significant difference in ALB content among all groups (*P* > 0.05) (Table 5).

### 3.5. Effects of Dietary Cholesterol Level on Digestive Enzyme Activities of *P. clarkii*

An increase in dietary cholesterol level shown significant quadratic trends in the activities of lipase and amylase in hepatopancreas and intestine, as well as in the protease in intestine (*P* < 0.05).

The maximum values of the activities of hepatopancreatic lipase and amylase were observed in group C4, which significantly increased by 8.79% and 50%, respectively, compared to group C0 (*P* < 0.05). No significant difference in the protease activity of the hepatopancreas was observed (*P* > 0.05). Group C3 showed the highest values for the activities of intestinal protease and amylase, which significantly increased by 31.48% and 236%, respectively, compared to group C0 (*P* < 0.05). The intestinal lipase activity of groups C2–C5 was significantly higher than that of group C0 (*P* < 0.05) (Table 6).

### 3.6. Effects of Dietary Cholesterol Level on Antioxidant Properties of the Hepatopancreas of *P. clarkii*

Increasing dietary cholesterol content resulted in quadratic trends in the T-AOC level and CAT activity in the hepatopancreas with statistical significance (*P* < 0.05). Additionally, both MDA contents and SOD activity in the hepatopancreas exhibited significant linear and quadratic trends (*P* < 0.05).

The highest values for T-AOC level, CAT activity, and SOD activity were observed in groups C3, C2, and C4, respectively, which were significantly greater than those in group C0 (*P* < 0.05). Compared to group C0, the MDA content was significantly lower in the groups with added cholesterol (*P* < 0.05) (Table 7).

### 3.7. Effects of Dietary Cholesterol Level on Hepatopancreas Tissue Structure of *P. clarkii*

**Figure 2:**
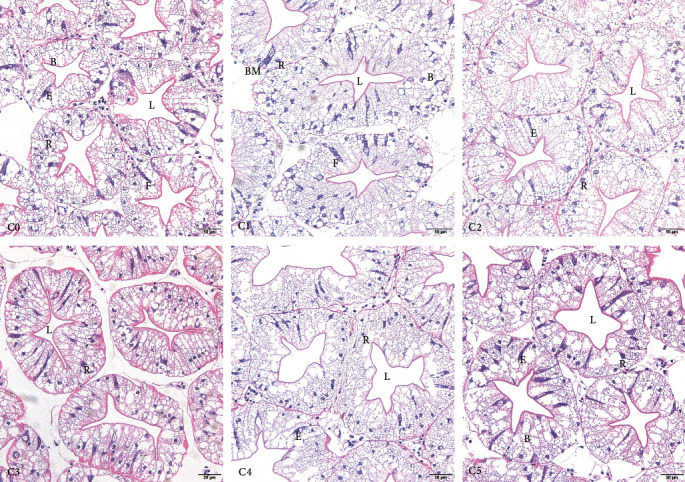
Effects of dietary cholesterol level on hepatopancreas microstructure of *P. clarkii*. C0–C5 represent each group; B, B cell; E, E cell; F, F cell; R, R cell; L, lumen; BM, basement membrane.

**Figure 3:**
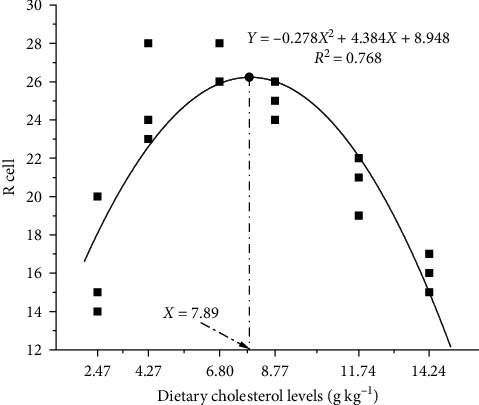
The quadratic regression analysis of R cells and dietary cholesterol level of *P. clarkii*.

The hepatopancreas and hepatic tubule lumen of *P. clarkii* were enlarged either by an excess (C5) or a deficiency (C0) of the dietary cholesterol. The group C2 exhibited a standard cell structure with clear cell boundaries. The maximum number of R cells was observed at a dietary cholesterol level of 7.89 g/kg (Figures 2 and 3).

## 4. Discussion

### 4.1. Effects of Dietary Cholesterol Level on Growth Performance of *P. clarkii*

The study found that *P. clarkii* exhibited the best growth performance when fed a diet supplemented with 10.49–10.93 g/kg cholesterol. This finding is consistent with studies conducted on *L. vannamei* [15], *Penaeus monodon* [25], and *L. spotted* [25]. In contrast, the cholesterol requirements of *L. vannamei* and *Penaeus japonicuswas* were found to be 1.90–2.70 g/kg [14] and 2.0–5.0 g/kg [12], respectively, which is lower than the present study finding. Other studies investigating *Macrobrachium rosenbergii* [26], *Penaeus porphyra* [24], and *Homarus americanus* [27] have reported that dietary cholesterol is not required. The variation in cholesterol requirements may be attributed to differences in the factors such as diet composition, feeding rate, water environment, rearing specifications, and feeding species. The main sources of dietary cholesterol are fish meal and fish oil. If dietary cholesterol is adequate, supplementation is unnecessary. High-plant-protein diet tends to be low in cholesterol, and crustaceans selectively absorb phytosterols. For instance, *β*-sitosterol is not efficiently absorbed by lobster [28]. Conversely, dietary lecithin and cholesterol exhibit a synergistic effect, enhancing the dissolution, transport, and utilization of cholesterol in crustaceans. For example, supplemental dietary cholesterol may not be essential for mud crab (*Scylla serrata*, *megalopa*) when fed diets containing adequate levels of the supplemental dietary phospholipids [29]. Therefore, fish meal and fish oil are not present or used in minimal proportions in *P. clarkii* feeds, supplements with high-cholesterol content, such as phospholipid oil, tallow, lard, poultry egg meal [30], butter, and phytosterol [14], can be added in moderate amounts to meet their growth requirements and reduce feed costs.

Molting is an essential aspect of crustacean growth and ensures proper muscle development. The present study showed that dietary cholesterol significantly improved the flesh percentage, similar to the effects of cholesterol injection on muofibier mass and size in the mice [31], indicating a potential role of cholesterol in promoting muscle growth. Based on the quadratic regression analysis of flesh percentage, the optimal cholesterol level for *P. clarkii* was 7.42 g/kg. This could be due to the fact that cholesterol is a precursor for the formation of ecdysteroids involved in the molting activity, and supplementing crustacean diets with appropriate amounts of cholesterol could play a role in reducing the incidence of molting death syndrome, increasing the frequency of molting, and promoting growth [32, 33].

### 4.2. Effects of Dietary Cholesterol Level on Proximate Composition and Lipids Content of *P. clarkii*

This study found that an appropriate amount of dietary cholesterol increased whole body protein content, PER and PDR, decreased hole body lipid content, similar results were found in *L. vannamei* [34], *Penaeus indicus* [35], and *Portunus trituberculatus* [36]. This suggests that an appropriate dietary cholesterol level may promote protein deposition and lipolysis, improving the utilization efficiency of feed protein and saving feed protein. In this study, the crude lipid content of the hepatopancreas increased with the dietary cholesterol level. This increase was attributed to the promotion of lipid synthesis and the inhibition of lipid degradation [37]. However, the specific molecular mechanism that led to this result requires further investigation.

Dietary cholesterol supplementation increased accumulation of triglyceride and total cholesterol in *L. vannamei* [38], *Epinephelus coioides* [39], *P. tridentatus* [36], and Chinese *E. sinensis* [37]. The cholesterol content was found to be higher in hepatopancreas than muscle or hemolymph, suggesting that cholesterol is primarily deposited in the hepatopancreas of *P. clarkii*. The positive correlation observed between hemolymph cholesterol content and dietary cholesterol level may stem from the dynamic mechanism of cholesterol utilization, wherein hemolymph cholesterol is transported to the hepatopancreas for storage as cholesterol esters when cholesterol requirements are not being fulfilled; muscle cholesterol levels do not increase until the requirements are met [37].

HDL-C primarily transports cholesterol from peripheral tissues to the liver for metabolism, while LDL-C has the opposite effect [2]. This study found an increase in hemolymph HDL-C content, while LDL-C initially increased and then decreased. These results indicated that dietary cholesterol can promote endogenous cholesterol metabolism in the shrimp up to a certain limit, but excessive cholesterol accumulation increases metabolic burden and results in poor growth performance [40, 41]. The hepatopancreas of *P. clarkii* contained significantly higher levels of free fatty acids than the hemolymph and muscle, which could indicate that fat is mainly stored in the hepatopancreas. Meanwhile, high-cholesterol intake triggers the self-cholesterol clearance mechanism, wherein cholesterol esterase breaks down into some extent of free fatty acids and cholesterol, which leads to an increase in cholesterol content and the content of free fatty acids [42].

### 4.3. Effects of Dietary Cholesterol Level on Digestive Performance of *P. clarkii*

The addition of 8.77 g/kg of cholesterol in this study resulted in a significant improvement of intestinal protease and lipase activities, which was comparable to the result obtained in *Cherax quadricarinatus* [43], indicating that dietary cholesterol promoted lipid and protein metabolism and absorption. This hypothesis was confirmed by the results of feed protein deposition rate and crude fat and crude protein contents of the whole shrimp. The hepatopancreas is the central organ of metabolism, digestion, absorption, storage, and excretion in crustaceans [44]. This study observed an initial increase followed by decrease in R cells, which corresponds to the observed trend in the free fatty acid content in both serum and muscle, as well as serum AST and ALT activities. As the storage of fatty acids increases in hepatopancreatic R cells, these cells will transport fatty acids to B cells for digestion and absorption, which will lead to an increase in vacuole size in the B cells. With a continuous increase in cholesterol intake, R cells exhibited a decreasing trend, indicating that a high dose of cholesterol may hinder lipid storage in the R cells. Moreover, both insufficient and excessive cholesterol supplementation resulted in an enlargement of the lumen of the hepatopancreas, suggesting that an inadequate or excessive intake of dietary cholesterol could harm the tissue structure of the hepatopancreas and cause hepatopancreatic dysfunction, and consuming an appropriate amount of cholesterol improved the health of the hepatopancreas in *P. clarkii*.

### 4.4. Effects of Dietary Cholesterol Level on Nonspecific Immunity of *P. clarkii*

Crustaceans are generally considered to rely on nonspecific immunity, owing to their weak specific immunity [45]. The oxidation state of crustacean organisms is typically measured using MDA, while T-AOC, CAT, and SOD are used as evaluation indices of antioxidant capacity [46]. This study found that cholesterol levels correlate with the fluctuation in activities of T-AOC, CAT, and SOD, which initially increase and then decrease. Simultaneously, the content of MDA showed a decreasing trend, which was similar to the results of *M. nipponensis* [15] and Chinese Mitten crab [10]. Total proteins in hemolymph include globulin and albumin, with globulin being linked to an immune response as is associated with the immune response [47]. In this study, there was no significant difference in albumin content, while the total protein content first increased and then decreased. It was speculated that the changing trend of globulin content was the same as that of total protein, indicating that the immune system was enhanced.

## 5. Conclusion

A diet that contains moderate cholesterol levels improved growth performance and feed utilization in *P. clarkia*. Specifically, by increasing protease and lipase activities, protein utilization efficiency is improved. In addition, muscle growth was promoted by regulating cholesterol stores, hepatopancreas function, and nonspecific immunity. According to a comprehensive analysis, the optimal dietary cholesterol level for *P. clarkii* was 7.42–10.93 g/kg.

## Figures and Tables

**Table 1:** Formulation and proximate composition of the experimental diets (dry matter basis except fat sources, g/kg).

Ingredients	Dietary cholesterol level (g/kg)					
	C0 (2.47)	C1 (4.27)	C2 (6.80)	C3 (8.77)	C4 (11.74)	C5 (14.24)
Fish meal	100	100	100	100	100	100
Soybean meal	150	150	150	150	150	150
Wheat gluten	100	100	100	100	100	100
Peanut meal	100	100	100	100	100	100
Flour	250	250	250	250	250	250
Corn starch	75	75	75	75	75	75
Fish oil	25	25	25	25	25	25
Corn oil	25	25	25	25	25	25
Microcellulose	48	45	42	39	36	33
Soybean lecithin	20	20	20	20	20	20
Cholesterol	0	3	6	9	12	15
Yeast extract	40	40	40	40	40	40
Sodium alginate	10	10	10	10	10	10
Calciumdihydrogen phosphate	28	28	28	28	28	28
Vitamin premix^1^	10	10	10	10	10	10
Mineral premix^2^	10	10	10	10	10	10
Allicin	1	1	1	1	1	1
Astaxanthin	1	1	1	1	1	1
Choline chloride	5	5	5	5	5	5
Dimethylsulfonium bromide	2	2	2	2	2	2
Proximate composition						
Dry matter	969.69	934.21	948.64	941.67	971.99	966.98
Crude protein	323.51	325.40	324.89	327.91	324.10	313.18
Crude lipid	78.87	79.66	86.11	87.00	94.12	95.92
Ash	69.08	63.85	66.27	65.66	69.63	69.17
Total energy (KJ/g)^3^	20.71	21.17	21.28	21.35	21.83	22.11

*Note*: C0–C5 represent each group. ^1^per kg of vitamin premix contains vitamin A 4 g, vitamin D 0.02 g, vitamin E 10 g, vitamin K3 10 g, vitamin B_1_ 10 g, vitamin B_2_ 10 g, vitamin B_6_ 20 g, nicotinic acid 40 g, biotin 0.2 g, calcium pantothenate 20 g, folic acid 0.5 g, vitamin B_12_ 0.01 g, vitamin C 20 g, and inositol 400 g, all ingredients were diluted with microcellulose to 1 kg. ^2^per kg of mineral premix contains KIO_3_ 0.6 g, Na_2_SeO_3_ · 5H_2_O 0.08 g, KH_2_PO_4_ 320 g, MgSO_4_ 200 g, MnSO_4_·H_2_O 20 g, CuCl_2_ · 2H_2_O 2 g, ZnSO_4_ · 7H_2_O 60 g, FeSO_4_ · 7H_2_O 50 g, NaCl 100 g, and CoCl_2_ · 6H_2_O 2 g, all ingredients were diluted with microcellulose to 1 kg. ^3^Energy was determined by direct combustion in an adiabatic bomb calorimeter (SDC311, Hunan Sundy Science and Technology Development Co., Ltd., Changsha, Hunan Province, China).

**Table 2:** Effect of dietary cholesterol level on the growth performance and feed utilization rate of *P. clarkii*.

Dietary cholesterol level (g/kg)	Parameters											
	IBW^1^ (g)	FBW^2^ (g)	WGR^3^ (%)	SGR^4^ (%/d)	FCR^5^	SR^6^ (%)	HSI^7^ (%)	AFC^8^ (%)	CFC^9^ (%)	TFC^10^ (%)	PER^11^ (%)	PDR^12^ (%)
2.47 (C0)	13.39 ± 0.47	21.35 ± 0.56^a^	59.47 ± 2.54^a^	0.83 ± 0.03^a^	1.50 ± 0.04^b^	88.89 ± 0.00	8.68 ± 0.59	12.41 ± 0.45	6.31 ± 0.07^b^	18.72 ± 0.38^a^	206.48 ± 0.64^a^	23.72 ± 1.72^a^
4.27 (C1)	13.45 ± 0.34	22.19 ± 0.85^ab^	64.97 ± 2.26^a^	0.90 ± 0.02^ab^	1.43 ± 0.06^b^	90.74 ± 8.49	9.22 ± 0.40	13.00 ± 0.90	6.41 ± 0.20^b^	19.41 ± 1.07^a^	220.10 ± 8.97^a^	25.34 ± 1.54^ab^
6.80 (C2)	13.53 ± 0.19	23.27 ± 0.57^bc^	71.95 ± 3.82^b^	0.97 ± 0.04^bc^	1.18 ± 0.06^a^	85.18 ± 3.21	9.29 ± 0.21	13.80 ± 0.66	7.79 ± 0.19^c^	21.59 ± 0.50^b^	249.64 ± 11.68^b^	27.79 ± 1.88^ab^
8.77 (C3)	13.51 ± 0.12	23.57 ± 0.52^c^	74.43 ± 2.33^bc^	0.99 ± 0.03^cd^	1.15 ± 0.05^a^	85.18 ± 3.21	8.84 ± 0.53	12.61 ± 0.41	6.82 ± 0.11^b^	19.43 ± 0.30^a^	259.39 ± 9.87^b^	28.61 ± 2.21^b^
11.74 (C4)	13.56 ± 0.07	24.52 ± 0.42^c^	80.87 ± 3.98^c^	1.06 ± 0.04^d^	1.16 ± 0.03^a^	88.89 ± 5.56	8.79 ± 0.83	12.52 ± 0.73	6.44 ± 0.12^b^	18.96 ± 0.82^a^	272.26 ± 7.15^b^	28.20 ± 2.22^ab^
14.24 (C5)	13.52 ± 0.08	23.33 ± 0.43^bc^	72.54 ± 2.90^b^	0.97 ± 0.03^bc^	1.21 ± 0.08^a^	79.63 ± 3.20	8.68 ± 0.26	12.08 ± 0.91	5.42 ± 0.41^a^	17.50 ± 1.26^a^	255.50 ± 15.07^b^	27.64 ± 0.14^ab^
ANOVA												
* P* value	0.967	0.001	0.001	0.001	0.001	0.128	0.546	0.125	0.001	0.001	0.001	0.033
Regression (*n* = 3)												
Linear												
Adj. *R*^2^	−0.015	0.506	0.543	0.534	0.509	0.183	−0.024	0.22	0.097	0.078	0.624	0.316
* P* value	0.401	0.001	0.001	0.001	0.001	0.043	0.447	0.256	0.112	0.138	0.001	0.009
Quadratic												
Adj. *R*^2^	−0.062	0.73	0.788	0.791	0.849	0.146	0.011	0.188	0.708	0.51	0.842	0.534
* P* value	0.615	0.001	0.001	0.001	0.001	0.12	0.36	0.082	0.001	0.002	0.001	0.001

*Note*: Values represent mean ± SD. Different letters in the same row indicate a significant difference between groups (*P* < 0.05). C0–C5 represent each group. *N*_*t*_ = the final mantissa; *N*_0_ = the initial mantissa; *W*_*t*_ = final body weight (g); *W*_0_ = the initial body weight (g); *t* = the experimental days (d); *W*_*f*_ = feed intake (g); *W*_*d*_ = the total dead crayfish weight (g); *W*_*h*_ = hepatopancreas weight (g); *W*_*m*_ = the muscle weight of shrimp tail (g); *Wc* = muscle weight of the first chelopod (g); *P* = the crude protein content of the feed (%); *P*_*t*_ = the final body crude protein content (%); *P*_*0*_ = the initial body protein content (%). ^1^IBW (g): initial mean weight. ^2^FBW (g): final mean weight. ^3^WGR (weight gain rate, %) = (*W*_*t*_ − *W*_0_)/*W*_0_ × 100. ^4^SGR (specific growth rate, %/d) = (ln*W*_*t*_ − ln*W*_0_)/*t* × 100. ^5^FCR (feed conversion ratio) = *W*_*f*_/(*W_t_N_t_* − *W*_0_*N*_0_ + *W*_*d*_). ^6^SR (survival rate, %) = *N*_*t*_/*N*_0_ × 100. ^7^HSI (hepatosomatic index, %) = *W*_*h*_/*W*_*t*_ × 100. ^8^AFC (abdomen flesh percentage, %) = *W*_*m*_/*W*_*t*_ × 100. ^9^CFC (clawfoot flesh percentage, %) = *W*_*c*_/*W*_*t*_ × 100. ^10^TFC (total flesh percentage, %) = (*W*_*m*_ + *W*_*c*_)/*W*_*t*_ × 100. ^11^PER (protein efficiency ratio, %) = (*W*_*t*_ − *W*_0_)/(*W*_*f*_ × *P*) × 100. ^12^PDR (protein deposition rate, %) = (*W*_*t*_ × *P*_*t*_ − *W*_0_ × *P*_0_)/(*W*_*f*_ × *W*_*p*_) × 100.

**Table 3:** Effects of dietary cholesterol level on the proximate composition of *P. clarkii*.

Dietary cholesterollevel (g/kg)	Whole body				Muscle				Hepatopancreas	
	Moisture	Crude protein	Crude lipid	Ash	Moisture	Crude protein	Crude lipid	Ash	Moisture	Crude lipid
2.47 (C0)	709.84 ± 18.47	120.15 ± 4.09^ab^	33.62 ± 0.17^e^	62.36 ± 7.62	791.18 ± 1.34	173.28 ± 3.55^a^	20.56 ± 0.30^e^	13.42 ± 0.12	571.33 ± 30.43	298.29 ± 17.81^a^
4.27 (C1)	707.38 ± 11.65	121.22 ± 6.66^ab^	32.33 ± 0.27^d^	63.92 ± 3.30	791.12 ± 2.96	175.21 ± 1.92^a^	19.71 ± 0.11^d^	13.60 ± 0.20	553.20 ± 75.71	313.50 ± 14.99^ab^
6.80 (C2)	709.53 ± 17.35	127.58 ± 5.54^b^	28.44 ± 0.50^b^	61.65 ± 2.53	793.40 ± 8.20	174.80 ± 3.36^a^	17.52 ± 0.13^b^	13.47 ± 0.27	546.3 ± 53.92	313.28 ± 11.62^ab^
8.77 (C3)	716.56 ± 13.13	118.69 ± 5.70^ab^	27.11 ± 0.19^a^	63.17 ± 4.93	787.97 ± 3.62	176.20 ± 3.42^a^	15.84 ± 0.10^a^	13.67 ± 0.90	552.83 ± 36.50	319.62 ± 18.49^abc^
11.74 (C4)	715.09 ± 18.65	117.09 ± 4.67^a^	29.69 ± 0.71^c^	61.25 ± 1.60	788.30 ± 3.25	177.83 ± 2.04^a^	18.74 ± 0.27^c^	13.76 ± 0.65	554.57 ± 20.88	339.45 ± 9.18^bc^
14.24 (C5)	710.19 ± 8.82	112.88 ± 7.08^a^	31.60 ± 0.40^d^	62.10 ± 3.02	782.63 ± 4.08	184.57 ± 3.38^b^	19.38 ± 0.15^d^	13.82 ± 0.51	525.90 ± 21.23	354.35 ± 6.08^c^
ANOVA										
* P* value	1.00	0.004	0.001	0.973	0.128	0.001	0.001	0.915	0.884	0.001
Regression (*n* = 3)										
Linear										
Adj. *R*^2^	−0.061	0.154	0.093	−0.049	0.261	0.49	0.033	0.028	0.017	0.104
* P* value	0.869	0.01	0.081	0.65	0.018	0.001	0.195	0.24	0.272	0.014
Quadratic										
Adj. *R*^2^	−0.129	0.257	0.874	−0.118	0.33	0.566	0.763	−0.036	−0.048	0.135
* P* value	0.973	0.003	0.001	0.905	0.019	0.001	0.001	0.509	0.556	0.001

*Note*: Values represent mean ± SD. Different letters in the same row indicate a significant difference between groups (*P* < 0.05).

**Table 4:** Effect of cholesterol level on lipids content of *P. clarkii*.

Parameters	Dietary cholesterol level (g/kg)						Regression (*n* = 3)				
							ANOVA	Linear		Quadratic	
	2.47 (C0)	4.27 (C1)	6.80 (C2)	8.77 (C3)	11.74 (C4)	14.24 (C5)	*P* value	Adj. *R*^2^	*P* value	Adj. *R*^2^	*P* value
Serum											
T-CHO (mmol/L)	0.155 ± 0.016^a^	0.164 ± 0.017^ab^	0.192 ± 0.026^ab^	0.201 ± 0.016^ab^	0.219 ± 0.035^bc^	0.256 ± 0.016^c^	0.001	0.731	0.001	0.729	0.001
TG (mmol/L)	0.31 ± 0.02^a^	0.38 ± 0.03^ab^	0.35 ± 0.08^ab^	0.36 ± 0.02^ab^	0.45 ± 0.05^b^	0.41 ± 0.02^ab^	0.02	0.163	0.054	0.16	0.106
HDL-C (mmol/L)	0.152 ± 0.013^a^	0.182 ± 0.009^b^	0.186 ± 0.006^b^	0.205 ± 0.002^c^	0.208 ± 0.004^c^	0.247 ± 0.004^d^	0.001	0.876	0.001	0.873	0.001
LDL-C (mmol/L)	0.099 ± 0.006^a^	0.100 ± 0.005^a^	0.106 ± 0.003^a^	0.118 ± 0.004^b^	0.119 ± 0.003^b^	0.108 ± 0.005^ab^	0.001	0.384	0.004	0.533	0.001
NEFA (mmol/L)	0.12 ± 0.01^bc^	0.13 ± 0.01^cd^	0.15 ± 0.01^df^	0.17 ± 0.00^f^	0.11 ± 0.01^ab^	0.09 ± 0.01^a^	0.001	0.129	0.079	0.701	0.001
Hepatopancreas											
T-CHO (mmol/g)	0.326 ± 0.012^a^	0.341 ± 0.011^ab^	0.345 ± 0.006^ab^	0.341 ± 0.005^ab^	0.365 ± 0.021^b^	0.350 ± 0.013^ab^	0.052	0.3	0.011	0.299	0.027
TG (mmol/g)	0.72 ± 0.05^a^	0.81 ± 0.07^ab^	0.82 ± 0.04^ab^	0.88 ± 0.02^b^	0.95 ± 0.07^bc^	1.01 ± 0.02^c^					
HDL-C (mmol/g)	0.194 ± 0.008^c^	0.162 ± 0.011^ab^	0.145 ± 0.013^a^	0.141 ± 0.003^a^	0.144 ± 0.013^a^	0.183 ± 0.001^bc^	0.001	0.008	0.301	0.83	0.001
LDL-C (mmol/g)	0.347 ± 0.015^b^	0.315 ± 0.020^b^	0.309 ± 0.017^b^	0.252 ± 0.006^a^	0.307 ± 0.020^b^	0.325 ± 0.018^b^	0.001	0.029	0.238	0.508	0.002
NEFA (*μ*mol/g)	6.44 ± 0.12^a^	6.79 ± 0.21^ab^	6.93 ± 0.28^ab^	6.70 ± 0.33^ab^	7.23 ± 0.36^b^	6.93 ± 0.14^ab^	0.047	−0.061	0.878	−0.122	0.928
Muscle											
T-CHO (mmol/g)	0.186 ± 0.019^a^	0.201 ± 0.010^a^	0.202 ± 0.012^a^	0.206 ± 0.010^a^	0.212 ± 0.006^a^	0.241 ± 0.013^b^	0.003	0.595	0.001	0.614	0.001
TG (mmol/g)	0.54 ± 0.04^a^	0.57 ± 0.05^a^	0.62 ± 0.03^ab^	0.64 ± 0.04^ab^	0.66 ± 0.03^b^	0.71 ± 0.05^b^					
HDL-C (mmol/g)	0.339 ± 0.023^a^	0.368 ± 0.036^ab^	0.382 ± 0.014^abc^	0.419 ± 0.015^bcd^	0.426 ± 0.005^cd^	0.439 ± 0.021^c^	0.001	0.761	0.001	0.763	0.001
LDL-C (mmol/g)	0.255 ± 0.007^ab^	0.251 ± 0.012^ab^	0.264 ± 0.005^b^	0.263 ± 0.005^b^	0.244 ± 0.002^a^	0.244 ± 0.020^a^	0.006	0.085	0.128	0.317	0.023
NEFA (*μ*mol/g)	0.38 ± 0.02^a^	0.41 ± 0.02^a^	0.68 ± 0.02^c^	0.65 ± 0.01^c^	0.65 ± 0.03^c^	0.60 ± 0.02^b^	0.001	0.103	0.105	0.638	0.001

*Note*: Values represent mean ± SD. Different letters in the same row indicate a significant difference between groups (*P* < 0.05). TCHO, total cholesterol; TG, triglyceride; LDL-C, high-density lipoprotein cholesterol; HDL-C, low-density lipoprotein cholesterol; NEFA, nonesterified fatty acids.

**Table 5:** Effect of cholesterol level on serum biochemical indexes of *P. clarkii*.

Dietary cholesterol level (g/kg)	TP (g/L)	ALB (g/L)	AST (U/L)	ALT (U/L)	GLU (mmol/L)
2.47 (C0)	66.87 ± 3.40^ab^	1.33 ± 0.06	8.00 + 1.00^a^	11.33 + 1.15^a^	0.41 ± 0.11
4.27 (C1)	68.91 ± 2.80^ab^	1.26 ± 0.14	10.00 + 1.00^ab^	17.33 + 1.15^b^	0.41 ± 0.09
6.80 (C2)	66.80 ± 4.93^ab^	1.42 ± 0.17	21.00 + 1.73^c^	21.00 + 1.00^bc^	0.43 ± 0.05
8.77 (C3)	77.72 ± 2.03^b^	1.47 ± 0.09	11.67 + 0.76^b^	21.33 + 1.15^c^	0.71 ± 0.10
11.74 (C4)	68.14 ± 6.53^ab^	1.36 ± 0.08	10.33 + 2.08^ab^	30.00 + 2.00^d^	0.76 ± 0.05
14.24 (C5)	62.33 ± 4.70^a^	1.40 ± 0.09	11.33 + 0.76^ab^	20.33 + 1.53^bc^	0.85 ± 0.12
ANOVA					
* P* value	0.02	0.86	0.058	0.087	0.038
Regression (*n* = 3)					
Linear					
Adj. *R*^2^	−0.048	−0.043	−0.06	0.231	0.483
* P* value	0.643	0.592	0.858	0.025	0.001
Quadratic					
Adj. *R*^2^	0.199	−0.073	0.064	0.307	0.461
* P* value	0.074	0.665	0.239	0.025	0.004

*Note*: Values represent mean ± SD. Different letters in the same row indicate a significant difference between groups (*P* < 0.05>). TP, total protein; ALB, albumin; AST, aminotransferase; ALT, alanine aminotransferase; GLU, glucose.

**Table 6:** Effect of cholesterol level on the hepatopancreas and intestinal digestive enzyme activities of *P. clarkii*.

Dietary cholesterol level (g/kg)	Hepatopancreas			Intestine		
	Protease (U/mg)	Lipase (U/g prot)	Amylase (U/mg prot)	Protease (U/mg)	Lipase (U/g prot)	Amylase (U/mg prot)
2.47 (C0)	72.56 ± 1.15	15.47 ± 0.19^a^	0.16 ± 0.03^ab^	26.33 ± 1.28^ab^	9.96 ± 0.37^a^	0.86 ± 0.02^a^
4.27 (C1)	70.22 ± 1.24	15.70 ± 0.36^ab^	0.14 ± 0.02^a^	27.61 ± 1.63^ab^	10.96 ± 0.85^ab^	1.37 ± 0.11^b^
6.80 (C2)	70.88 ± 2.35	16.07 ± 0.33^bc^	0.15 ± 0.01^a^	29.24 ± 1.81^b^	11.41 ± 0.52^b^	2.35 ± 0.14^c^
8.77 (C3)	72.16 ± 1.40	16.39 ± 0.07^cd^	0.20 ± 0.01^bc^	34.62 ± 1.32^c^	11.60 ± 0.13^b^	2.89 ± 0.06^d^
11.74 (C4)	70.29 ± 3.46	16.83 ± 0.05^d^	0.24 ± 0.02^c^	26.18 ± 1.27^ab^	11.77 ± 0.44^b^	2.86 ± 0.08^d^
14.24 (C5)	72.69 ± 1.67	16.82 ± 0.12^d^	0.22 ± 0.01^c^	24.75 ± 1.41^a^	11.95 ± 0.33^b^	2.84 ± 0.16^d^
ANOVA						
* P* value	0.334	0.001	0.001	0.001	0.001	0.001
Regression (*n* = 3)						
Linear						
Adj. *R*^2^	−0.041	0.859	0.582	−0.053	0.648	0.81
* P* value	0.774	0.001	0.001	0.702	0.001	0.001
Quadratic						
Adj. *R*^2^	−0.015	0.855	0.573	0.431	0.73	0.952
* P* value	0.45	0.003	0.001	0.006	0.001	0.001

*Note*: Values represent mean ± SD. Different letters in the same row indicate a significant difference between groups (*P* < 0.05).

**Table 7:** Effects of cholesterol level on antioxidant capacity of hepatopancreas of *P. clarkii*.

Dietary cholesterol level (g/kg)	T-AOC (mmol/g prot)	CAT (U/mg prot)	SOD (U/mg prot)	MDA (nmol/mg prot)
2.47 (C0)	1.24 ± 0.09^a^	13.04 ± 1.34^a^	65.96 ± 4.88^a^	13.23 ± 0.39^b^
4.27 (C1)	1.51 ± 0.02^b^	19.03 ± 0.71^bc^	67.72 ± 3.16^a^	11.03 ± 0.34^a^
6.80 (C2)	1.53 ± 0.05^b^	21.09 ± 1.47^c^	68.03 ± 3.29^a^	10.55 ± 0.58^a^
8.77 (C3)	1.71 ± 0.06^c^	20.34 ± 0.78^c^	70.17 ± 3.35^a^	10.65 ± 0.66^a^
11.74 (C4)	1.18 ± 0.04^a^	17.06 ± 0.70^b^	78.78 ± 3.11^b^	10.41 ± 0.53^a^
14.24 (C5)	1.15 ± 0.03^a^	12.05 ± 0.80^a^	71.70 ± 2.91^ab^	9.68 ± 0.72^a^
ANOVA				
* P* value	0.001	0.001	0.001	0.001
Regression (*n* = 3)				
Linear				
Adj. *R*^2^	0.028	−0.036	0.341	0.613
* P* value	0.241	0.529	0.002	0.001
Quadratic				
Adj. *R*^2^	0.649	0.927	0.332	0.694
* P* value	0.001	0.001	0.006	0.001

*Note*: Values represent mean ± SD. Different letters in the same row indicate a significant difference between groups (*P* < 0.05). T-AOC, total antioxidant capacity; CAT, catalase; SOD, superoxide dismutase; MDA, malondialdehyde.

## Data Availability

All data supporting this research article are available from the corresponding author on request.
